# InfoMSD: an information-maximization self-distillation framework for parameter-efficient fine-tuning on artwork images

**DOI:** 10.3389/frai.2026.1721866

**Published:** 2026-03-04

**Authors:** Feng Guan, Hao Hong, Yong Wang

**Affiliations:** 1 School of Mathematics and Statistics, Southwest University, Chongqing, China; 2 Postdoctoral Research Center, Beijing Pico Exhibition Management Co. Ltd., Beijing, China

**Keywords:** artwork recognition, information-maximization, parameter-efficient fine-tuning, self-distillation, vision-language models

## Abstract

In recent years, despite the remarkable performance of large-scale vision language models across various visual classification tasks, their substantial parameter counts and high fine-tuning costs have hindered deployment in resource-constrained cultural and artwork settings. This work specifically addresses the task of object recognition in artwork—that is, identifying semantic objects (e.g., animals, people, everyday items) depicted within paintings, sketches, and other artistic renditions, rather than classifying artistic styles or genres. To address this issue, we propose InfoMSD, an unsupervised, Information-Maximization Self-Distillation framework designed for parameter-efficient fine-tuning on unlabeled artwork imagery while preserving robust performance. Specifically, InfoMSD incorporates a teacher-student architecture in the self-distillation phase, where the teacher model generates pseudo-labels for artworks, and the student model learns from the teacher through cross-entropy. By aligning the student's predictions with the discriminative signals from the teacher's pseudo-labels and simultaneously applying entropy-based regularization to sharpen the probability distribution and balance class coverage, the framework improves both the quality of the pseudo-labels and the discriminative capacity of the model. To enable parameter-efficient fine-tuning, only the layer norm parameters and visual prompts in the student model are updated, while the remaining parameters are frozen, significantly reducing computational overhead. Extensive experimental results on artwork datasets show that InfoMSD achieves accuracy improvements of +6.43 and +3.02% over CLIP zero-shot baselines, while adjusting less than 1% of the model parameters. Compared to existing lightweight distillation methods, InfoMSD achieves average accuracy gains of 1.35 and 0.96%, respectively. Overall, InfoMSD offers a novel, information-theoretic paradigm for unsupervised and efficient fine-tuning in object recognition within artistic imagery, balancing performance and efficiency.

## 1 Introduction

Object recognition in artwork plays a vital role in preserving, interpreting, and disseminating cultural heritage. Unlike artwork style classification (e.g., distinguishing Impressionism from Baroque), this task focuses on identifying the semantic content—such as objects, animals, and human figures—depicted within artistic images including paintings, sketches, and renditions. As cultural heritage institutions worldwide embark on digitization, there is a growing need to develop intelligent systems that can classify and explain visual collections (Li et al., 2024). It is worth noting that while fine art museums may prioritize style-based classification (e.g., Impressionism, Baroque) or brush texture analysis, content-based object recognition remains valuable for diverse cultural institutions—such as natural history museums, archaeological archives, and illustrated manuscript collections—where identifying depicted subjects (e.g., species, artifacts, historical figures) is essential for cataloging and retrieval. The manual process of organizing textual information, such as exhibit descriptions, relies on the subjective judgments of curators or volunteers. This method is time-consuming and lacks uniform standards for classification and naming styles (Mohan and Rodgers, 2021). There are inconsistencies in the use of categorization terms and labels for similar exhibits between different museums or even between different artwork within the same museum. Such unstructured exhibit descriptions constrain the digital integration and reuse of cultural heritage (King et al., 2023). In addition, these classification methods are difficult to adapt to the rapid updating needs of large-scale data collection scenarios. Moreover, manual proofreading and categorization are prone to omissions, errors, and subjective bias (Yang et al., 2023).

In the task of artwork classification, we introduce two complementary explainability techniques to enhance the interpretability of decisions made by vision-language models (VLMs): multi modal saliency analysis and embedding-space visualization.

For saliency analysis, instead of using traditional methods such as Gradient-weighted Class Activation Mapping (Grad-CAM) (Selvaraju et al., 2017), Local Interpretable Model-agnostic Explanations (LIME) (Ribeiro et al., 2016), or Shapley Additive Explanations (SHAP) (Mosca et al., 2022), we adopt the state-of-the-art multi modal explanation technique MM-SHAP (Performance-agnostic Metric for Measuring Multimodal Contributions) (Parcalabescu and Frank, 2023). MM-SHAP enables fine-grained attribution of both visual regions and textual keywords in the joint embedding space, thus providing more precise insights into the model's decision-making process. This approach significantly improves semantic interpretability, especially for complex categories such as “goldfish sketch” and “goldfish oil painting.” By leveraging dual-modality attribution, MM-SHAP offers interpretable feedback that is particularly valuable for curators and art historians.

To further understand how model representations evolve during adaptation, we employ t-distributed Stochastic Neighbor Embedding (t-SNE) to visualize the distribution of learned features. The t-SNE projections clearly illustrate the progressive clustering and separation of different artistic categories in the embedding space as training progresses. This provides visual evidence for the model's performance improvement and reveals its internal learning dynamics (Ribeiro et al., 2016).

However, conventional explanation methods often suffer from substantial computational overhead and latency, which limits their applicability in large-scale, multi modal scenarios like museum environments that demand high-throughput or real-time interpretation (Ezeji et al., 2024). To address these challenges, we combine MM-SHAP-based saliency analysis with efficient clustering visualization, offering a practical and interpretable solution for real-world artwork classification tasks.

The contributions of this paper are summarized as follows:

- Propose an information maximization framework that addresses the domain gap between natural images and artwork for efficient semantic object recognition in artistic imagery, requiring minimal computational overhead compared to existing methods.
- Develop an enhanced label-free tuning approach that overcomes LaFTer's limitations by integrating VLM adaptation with information maximization, achieving better feature clustering and classification performance with reduced training costs.

The remainder of this paper is structured as follows. Section 2 reviews the relevant literature. Section 3 introduces the method we use in the experimental procedure. Section 4 introduces the experimental setup and presents the results that demonstrate the effectiveness of the framework. Section 5 discusses the limitations of the current approach and explains why multi-modal information matters for painting recognition. Finally, Section 6 concludes the paper and outlines directions for future work.

## 2 Related works

### 2.1 Vision-language models (VLMS)

Vision-language models have become a powerful approach for understanding the relationship between visual and textual information. Contrastive Language-Image Pre-training (CLIP) (Radford et al., 2021) is a key work in this area. It shows strong zero-shot capabilities by learning visual concepts from natural language supervision. CLIP uses contrastive learning to align image and text representations in a shared space. This allows zero-shot classification by computing similarity between image features and text embeddings of class names. Many works have built on CLIP's success to improve performance in different scenarios. These models work well for domain adaptation tasks. The rich semantic knowledge learned from large-scale web data can transfer to specialized domains. VLMs can understand both visual content and text descriptions. This makes them suitable for cultural heritage applications like artwork analysis, where objects may have historical or artistic significance.

### 2.2 Parameter efficient fine-tuning

Full fine-tuning of large pre-trained models is computationally expensive and risky. Parameter efficient methods have gained attention as alternatives. These approaches adapt pre-trained models to new tasks while updating only a small subset of parameters.

Prompt learning approaches: Context Optimization (CoOp) (Zhou et al., 2022a) introduces learnable prompts for vision-language models. Additionally, in Visual Prompt Tuning (VPT) (Jia et al., 2022) a set of learnable visual prompt embeddings is prepended to the model's input embedding space and concatenated with the original image embeddings before being fed into the model. Optimizing context vectors while keeping the pre-trained model frozen shows that learning continuous prompts significantly improves performance over hand-crafted prompts in various vision tasks (Lu et al., 2025b).

Adapter-based methods: it contains CLIP-Adapter (Gao et al., 2023) introduces lightweight adapter modules for pre-trained CLIP models. These adapters use bottleneck layers that learn task-specific transformations. They preserve the general knowledge of the pre-trained model. This approach enables efficient adaptation to new domains with minimal parameter updates (Lu et al., 2025d). LP++ (Huang et al., 2024) improves upon a simple linear probe by augmenting text features and calibrating the final classifier's weights with zero-shot statistics. While surprisingly effective for few-shot classification, its reliance on labeled data for training the probe makes it unsuitable for purely unsupervised settings.

Additive fine-tuning: LoRA (Hu et al., 2022) significantly reduces the number of trainable parameters and storage overhead by injecting trainable, paired low-rank decomposition matrices into specific model layers, while keeping the pre-trained weights frozen. In addition, some studies have shown that additionally fine-tuning the Layer Normalization (LN) parameters can further stabilize and enhance model performance, as these layers play a crucial role in governing layer activations and feature distributions (Houlsby et al., 2019, Lu et al., 2025a).

### 2.3 Domain adaptation

Domain adaptation tackles the challenge of transferring knowledge from a source domain to a target domain with different data distributions. This is important for specialized domains like art and cultural heritage. Visual characteristics in these domains may differ substantially from natural images used in pre-training.

Knowledge distillation for domain generalization: knowledge distillation has emerged as a promising paradigm for enhancing domain generalization by transferring robust representations from teacher to student networks. KDDG (Wang et al., 2021) introduces a regularized knowledge distillation framework that leverages “dark knowledge” from teacher networks combined with a gradient filter to improve cross-domain generalization. The method demonstrates that richer supervisory signals from teachers can reduce the difficulty of learning domain-invariant mappings. More recently, XDED (Lee et al., 2022) proposes cross-domain ensemble distillation, which promotes convergence to flat minima in the loss landscape, thereby improving generalization to unseen domains while accelerating the training process. Both methods have been extensively evaluated on multi-domain benchmarks such as PACS and OfficeHome, establishing strong baselines for distillation-based domain generalization. Our InfoMSD framework shares the teacher-student distillation philosophy but differs in two key aspects: (1) we operate in a label-free setting without requiring ground-truth annotations, and (2) we integrate information maximization objectives to ensure confident yet diverse predictions, which is particularly beneficial for artistic imagery where class boundaries are often ambiguous.

Label-free adaptation: within this paradigm, two primary strategies have emerged. One approach is pseudo-labeling, as exemplified by POUF (Tanwisuth et al., 2023). It leverages the strong zero-shot capability of the pre-trained model to generate initial pseudo-labels for an unlabeled target dataset, which are then used for self-supervised training of prompt vectors. Another strategy relies on consistency regularization, as demonstrated by LaFTer (Mirza et al., 2023). This method learns by enforcing representation consistency between different augmented views of the same unlabeled image, often guided by language to prevent model collapse.

Prior-based enhancement: CLIP with Priors (CLIP-PR) (Li et al., 2023) incorporates label distribution priors to enhance zero-shot classification performance. Different domains may have varying class distributions. This approach leverages this knowledge to improve classification accuracy (Lu et al., 2025c).

Test-time adaptation: in this paradigm, a model is adapted on-the-fly using the unlabeled test data itself to handle potential distribution shifts. TDA (Liu et al., 2024) presents an efficient approach, fine-tuning a lightweight adapter through entropy minimization of predictions on the test batch. This self-supervised signal incentivizes more confident and consistent predictions, thus improving zero-shot performance without labels. Boost Adapter (Zhang et al., 2024) aims to enhance robustness via a regional bootstrapping mechanism, which leverages region proposals to identify diverse semantic parts of an image rather than relying on predictions from the entire image.

The combination of these approaches provides a foundation for developing robust models that can recognize objects in artwork. They leverage the strengths of pre-trained vision-language models while adapting to the specific characteristics of the artistic domain through efficient and unsupervised methods.

**Figure 1:**
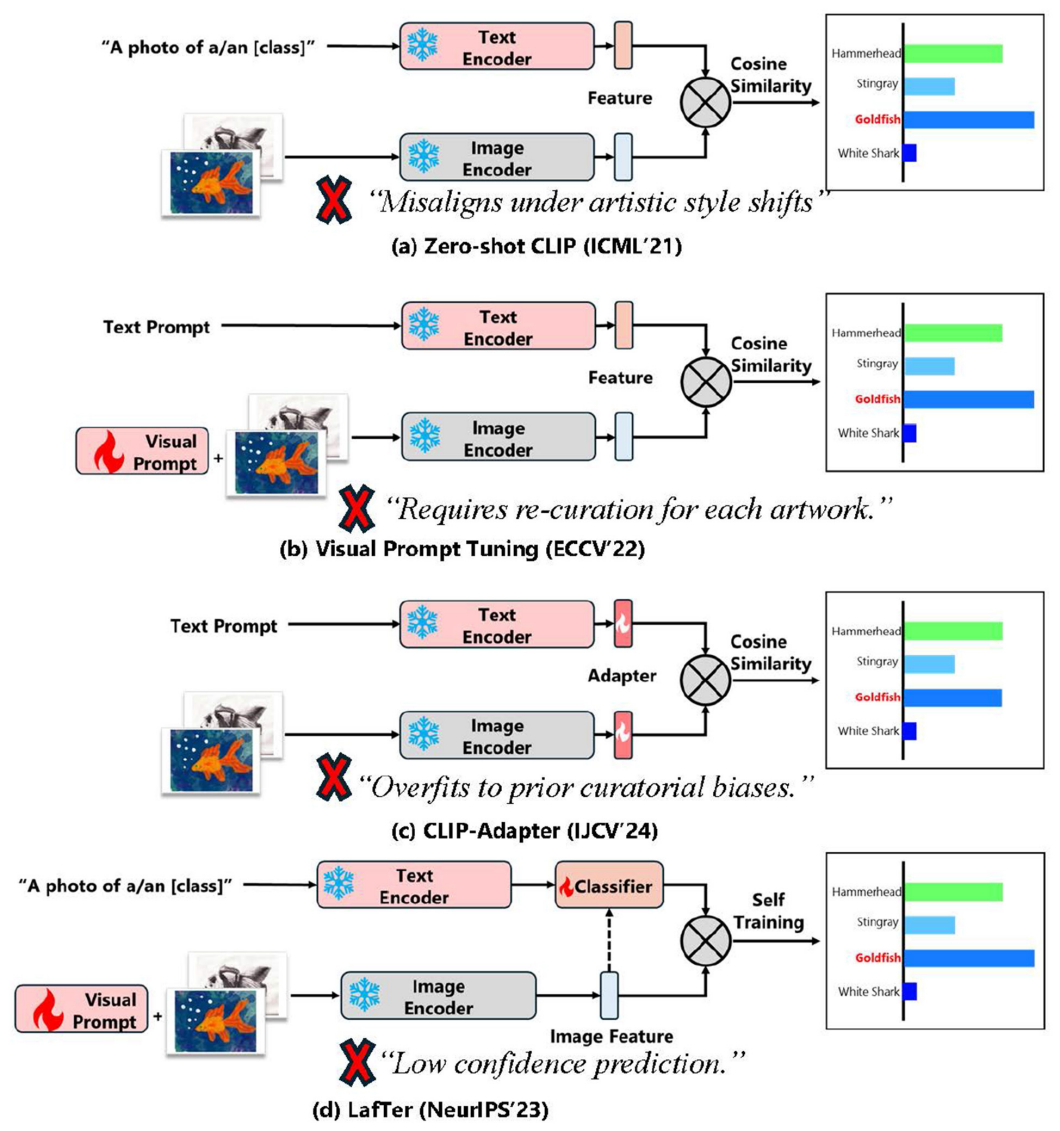
Comparison of prior CLIP-based adaptation methods on artistic domain. **(a)** Zero-shot CLIP suffers from poor alignment under artistic style shifts. **(b)** Visual Prompt Tuning adapts with shallow features but overfit to prior curatorial biases. **(c)** CLIP-Adapter requires re-curation for each artwork. **(d)** LaFTer overlooks artistic information maximization, leading to low-confidence predictions.

Figure 1 illustrates the limitations of representative CLIP-based adaptation techniques when applied to artwork. Zero-shot CLIP struggles with domain misalignment under artistic styles. Visual prompt tuning introduces adaptability but risks overfitting to existing curatorial biases. CLIP-Adapter requires manual re-curation for every artwork context, while LaFTer neglects uncertainty modeling, resulting in low-confidence predictions.

## 3 Method

### 3.1 Contrastive language-image pre-training

Contrastive Language-Image Pre-training (CLIP) (Radford et al., 2021) has demonstrated remarkable zero-shot classification capabilities on natural images. However, it faces substantial challenges when applied to specialized domains such as artwork. The core issue stems from the distributional shift between CLIP's pre-training data and the artistic domain.

To be precise, CLIP learns from a large-scale source domain $S={\left\{\left(I_{i},T_{i}\right)\right\}}_{i=1}^{N}$ of natural images and their corresponding textual descriptions. It consists of a visual encoder $\phi_{v}:{\mathbb{R}}^{H\times W\times C}\to {\mathbb{R}}^{d}$ and a text encoder $\phi_{t}:V\to {\mathbb{R}}^{d}$ that map images and text from their native spaces into a unified *d*-dimensional embedding space. For a given target image *x* and a set of *K* class labels, the standard zero-shot classification probability is computed as follows. First, a set of text prompts $T=\left\{T_{1},\ldots ,T_{K}\right\}$ is created (e.g., “a photo of a {class_k}”). The model then extracts the image feature *v* = ϕ_*v*_(*x*) and the corresponding text features {*t*_1_, …, *t*_*K*_}, where each *t*_*k*_ = ϕ_*t*_(*T*_*k*_). The probability *p*(*y* = *k*|*x*) is then given by:


$$\begin{array}{l} p\left(y=k|x\right)=\frac{\exp\left(\text{sim}\left(v,t_{k}\right)/\tau \right)}{\overset{K}{\underset{i=1}{\sum }}\exp\left(\text{sim}\left(v,t_{i}\right)/\tau \right)} \end{array}$$ (1)

where sim(·, ·) denotes the cosine similarity, and τ is a trainable temperature parameter learned during pre-training to scale the logits. Intuitively, this equation computes a softmax over the similarity scores between the image and all class descriptions: the higher the similarity between an image feature *v* and a text feature *t*_*k*_, the higher the probability that the image belongs to class *k*. The temperature τ controls the “sharpness” of the distribution—smaller values produce more confident (peaky) predictions, while larger values yield softer (more uniform) distributions.

This aforementioned distributional shift manifests in three fundamental limitations when applying the model to artwork $P={\left\{P_{j}\right\}}_{j=1}^{M}$: (1) *stylistic divergence*, as the visual features optimized for photographic content poorly capture artistic techniques, brushwork patterns, and color compositions; (2) *temporal displacement*, because objects in historical paintings appear in anachronistic contexts absent from contemporary training data; (3) *artistic variance*, given that the same semantic object exhibits dramatically different visual manifestations across artistic movements and individual styles. Our goal is to develop a robust classifier for object recognition in paintings P across target categories K without requiring manual annotation of artistic content.

### 3.2 Self-distillation adaptation

We propose a two-stage adaptation framework designed to enhance model performance on the target domain. The framework first utilizes a zero-shot classifier as a *teacher* to generate pseudo-labels from unlabeled data. Subsequently, a *student* model, augmented with parameter-efficient modules, learns from these labels via a self-distillation process guided by consistency regularization. Given the set of unlabeled paintings P, a teacher model is first used to generate high-quality pseudo-labels. For each painting P∈P, we create a weakly augmented view $I_{t}=A_{\text{weak}}\left(P\right)$. The teacher model then produces a “hard” pseudo-label Ỹ(*P*) by assigning the class with the highest confidence:


$$\begin{array}{l} \tilde{Y}\left(P\right)=\text{one\_hot}\left(\arg\underset{k\in K}{\max}h_{t}{\left(\phi_{v}\left(I_{t}\right)\right)}_{k}\right) \end{array}$$ (2)

Here, *h*_*t*_ denotes the teacher's classifier prototypes, generated by passing text prompts through the CLIP text encoder. In plain terms, the teacher model examines a painting and assigns it to the single class with the highest confidence score. The argmax operation selects this winning class, and one_hot converts it into a vector of zeros with a single one at the winning class position (e.g., [0, 0, 1, 0, …] if class 3 is selected). This “hard” pseudo-label then serves as the training target for the student model. These prompts can either be standard, hand-crafted templates (e.g., “a photo of a {class_*k*}”) or ensemble prompts generated by a Large Language Model (LLM), like LaFTer (Mirza et al., 2023).

The student model is designed for efficient adaptation by incorporating learnable visual prompts. For the same painting *P*, we generate a strongly augmented view, which is then processed by the visual encoder's embedding layer to produce patch embeddings *E*∈ℝ^*N*×*d*^ by $A_{\text{stong}}\left(P\right)$. We then prepend a set of trainable prompt tokens $V=\left\{v_{1},\ldots ,v_{L}\right\}\in {\mathbb{R}}^{L\times d}$ to these patch embeddings. The final input sequence for the student's vision transformer, denoted as *I*_*s*_, is formed by this concatenation:


$$\begin{array}{l} I_{s}=\text{Concat}\left(E_{\text{cls}},V,E\right) \end{array}$$ (3)

Here, *E*_cls_ is the special classification token, V represents the learnable visual prompts (additional “hint” tokens that the model can adjust during training), and *E* contains the patch embeddings extracted from the input image. By concatenating these components, we inject trainable parameters into the input sequence without modifying the frozen backbone, enabling parameter-efficient adaptation.

The student's prediction, a probability distribution ŷ(*P*), is then obtained by passing *I*_*s*_ through the transformer layers and the student's classifier head:


$$\begin{array}{l} \hat{y}\left(P\right)=h_{s}\left(\phi_{v}\left(I_{s}\right)\right) \end{array}$$ (4)

The adaptation objective is to minimize the discrepancy between the teacher's pseudo-labels and the student's predictions using a cross-entropy loss:


$$\begin{array}{l} L_{adapt}=-\overset{K}{\underset{k=1}{\sum }}\tilde{Y}{\left(P\right)}_{k}\log\left(\hat{y}{\left(P\right)}_{k}\right) \end{array}$$ (5)

This is the standard cross-entropy loss commonly used in classification tasks. Since Ỹ(*P*) is a one-hot vector, the summation effectively reduces to −log(ŷ(*P*)_*c*_), where *c* is the pseudo-labeled class. Minimizing this loss encourages the student to assign high probability to the class predicted by the teacher. The closer the student's prediction is to the teacher's pseudo-label, the lower the loss value.

Crucially, during this process, only the visual prompts V, the LayerNorm parameters of the vision encoder, and the student's classifier parameters *h*_*s*_ are updated. This ensures parameter efficiency, with less than 1% of the total model parameters being trainable.

### 3.3 Information maximization for student adaptation

To further guide the student model's adaptation and mitigate the inherent uncertainty in pseudo-labeling, we incorporate an information maximization objective. This principle encourages the model to produce confident predictions for individual samples while ensuring a diverse distribution of predictions across the entire dataset. This is achieved by optimizing two complementary entropy-based losses, which are computed over a mini-batch of paintings B⊂P.

First, we define a conditional entropy loss, $L_{\text{ent}}$, which encourages the student model to be decisive for each painting in the batch. It is calculated as the average entropy of predictions:


$$\begin{array}{l} L_{\text{ent}}=-\frac{1}{\left|B\right|}\underset{P\in B}{\sum }\overset{K}{\underset{k=1}{\sum }}{\hat{y}}_{k}\left(P\right)\log{\hat{y}}_{k}\left(P\right) \end{array}$$ (6)

where ŷ_*k*_(*P*) is the *k*-th element of the student's predicted probability vector ŷ(*P*). Minimizing this loss pushes the predictions toward one-hot vectors, indicating high confidence. To understand this intuitively: entropy measures uncertainty. A prediction like [0.5, 0.5] (completely uncertain between two classes) has high entropy, while [0.99, 0.01] (confident prediction) has low entropy. By minimizing this term, we encourage the model to “make up its mind” and produce decisive predictions rather than hedging across multiple classes.

Second, to prevent the model from collapsing to a few classes (mode collapse), we introduce a diversity loss, $L_{\text{div}}$. This loss maximizes the entropy of the average prediction across the batch:


$$\begin{array}{l} L_{\text{div}}=-\overset{K}{\underset{k=1}{\sum }}{ȳ}_{k}\log{ȳ}_{k},\;\text{where }ȳ=\frac{1}{\left|B\right|}\underset{P\in B}{\sum }\hat{y}\left(P\right) \end{array}$$ (7)

Here, ȳ is the average prediction vector for the mini-batch. Maximizing this term encourages the model to utilize all available classes. Consider a failure case: if the model predicts every image as “goldfish,” the average prediction ȳ would be [1, 0, 0, …], which has zero entropy. By maximizing $L_{\text{div}}$, we push ȳ toward a uniform distribution (e.g., [0.1, 0.1, …] for 10 classes), ensuring the model spreads its predictions across all classes rather than collapsing to a single dominant class. This prevents the well-known “mode collapse” problem in unsupervised learning.

The complete information maximization objective is a weighted combination of these two terms:


$$\begin{array}{l} L_{\text{info}}=L_{\text{ent}}-L_{\text{div}} \end{array}$$ (8)

This regularization ensures that the adapted model maintains its discriminative capabilities while avoiding overconfident predictions on ambiguous artistic content.

The two terms work in complementary directions: minimizing $L_{\text{ent}}$ makes individual predictions confident (low entropy per sample), while maximizing $L_{\text{div}}$ (equivalent to minimizing $-L_{\text{div}}$) ensures diverse predictions across the batch (high entropy of average predictions). Together, they embody the principle of information maximization: the model should be certain about each individual prediction while maintaining uncertainty about which class will appear next in the dataset.

### 3.4 InfoMSD

Our complete training objective combines all components:


$$\begin{array}{l} L_{total}=L_{adapt}+\lambda L_{info}=L_{adapt}+\lambda \left(L_{\text{ent}}-L_{\text{div}}\right) \end{array}$$ (9)

where λ controls the contribution of information maximization. The optimization alternates between pseudo-label generation and model parameter updates, with information maximization serving as a regularization mechanism that promotes robust adaptation across diverse artistic styles.

The resulting framework effectively bridges the domain gap between natural photographs and artistic paintings while maintaining computational efficiency and avoiding the need for manual annotation. The integration of information maximization ensures stable adaptation performance across the varied visual characteristics inherent in artwork.

Figure 1 illustrates the limitations of representative CLIP-based adaptation techniques when applied to artwork. Zero-shot CLIP struggles with domain misalignment under artistic styles. Visual prompt tuning introduces adaptability but risks overfitting to existing curatorial biases. CLIP-Adapter requires manual re-curation for every artwork context, while LaFTer neglects uncertainty modeling, resulting in low-confidence predictions.

**Figure 2:**
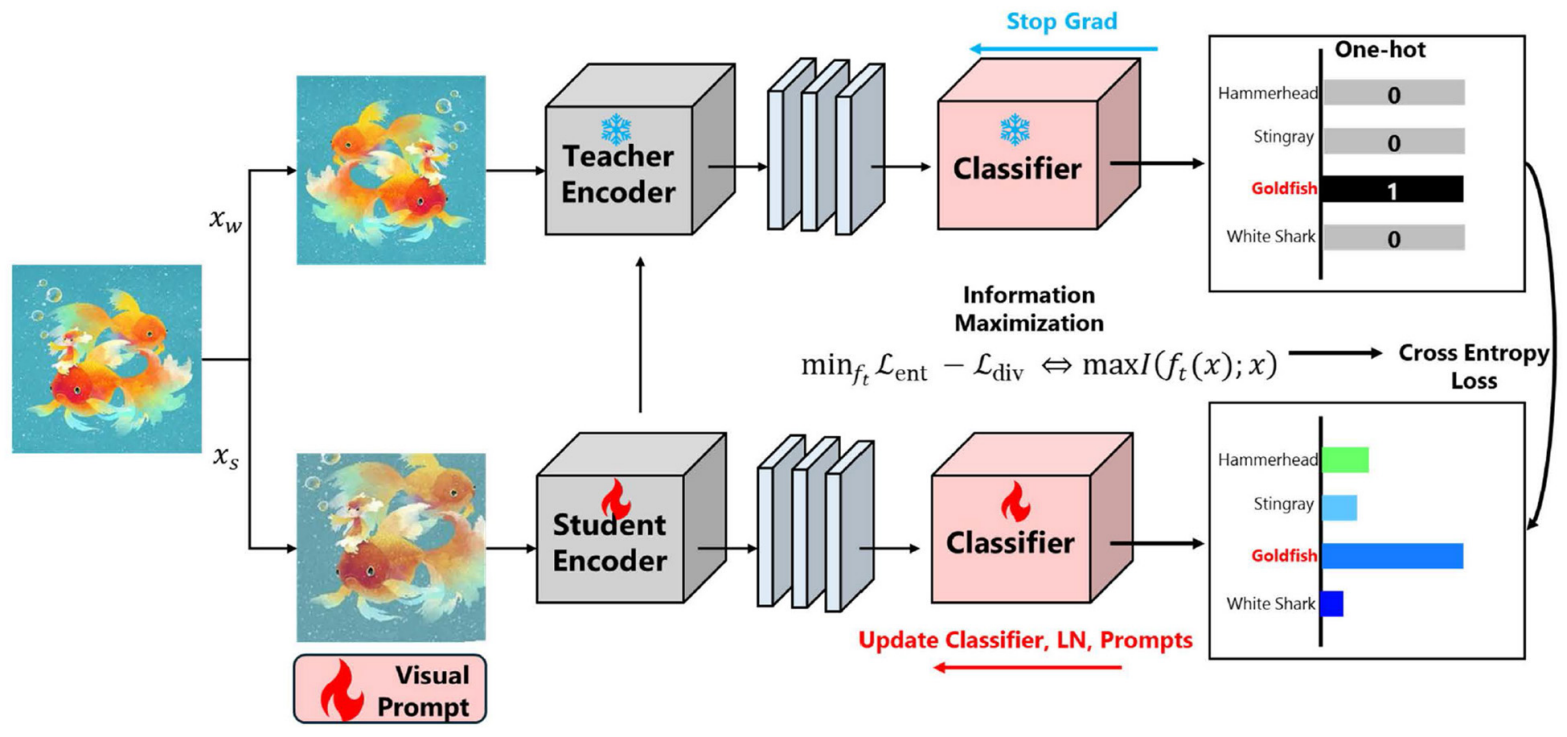
Proposed framework overview. The model leverages synthetic text generation, prompt tuning, and mutual information maximization to achieve label-free adaptation for artwork.

To address these challenges, Figure 2 presents our proposed framework, which integrates synthetic text generation, pseudo-label-driven prompt tuning, and information-theoretic-base regularization. This approach ensures robust adaptation to diverse artistic styles without relying on labeled training data from the target domain.

To facilitate reproducibility, we provide a step-by-step summary of the complete InfoMSD training procedure in Algorithm 1. The algorithm consists of four main steps per iteration: (1) the teacher generates hard pseudo-labels using weak augmentations, (2) the student processes strongly augmented images with learnable visual prompts, (3) the total loss combines adaptation and information maximization objectives, and (4) only the parameter-efficient modules are updated via backpropagation. Note that unlike some self-distillation methods, our teacher remains fixed (no exponential moving average update) to maintain stable pseudo-label quality throughout training.

Algorithm 1 InfoMSD: information-maximization self-distillation. 
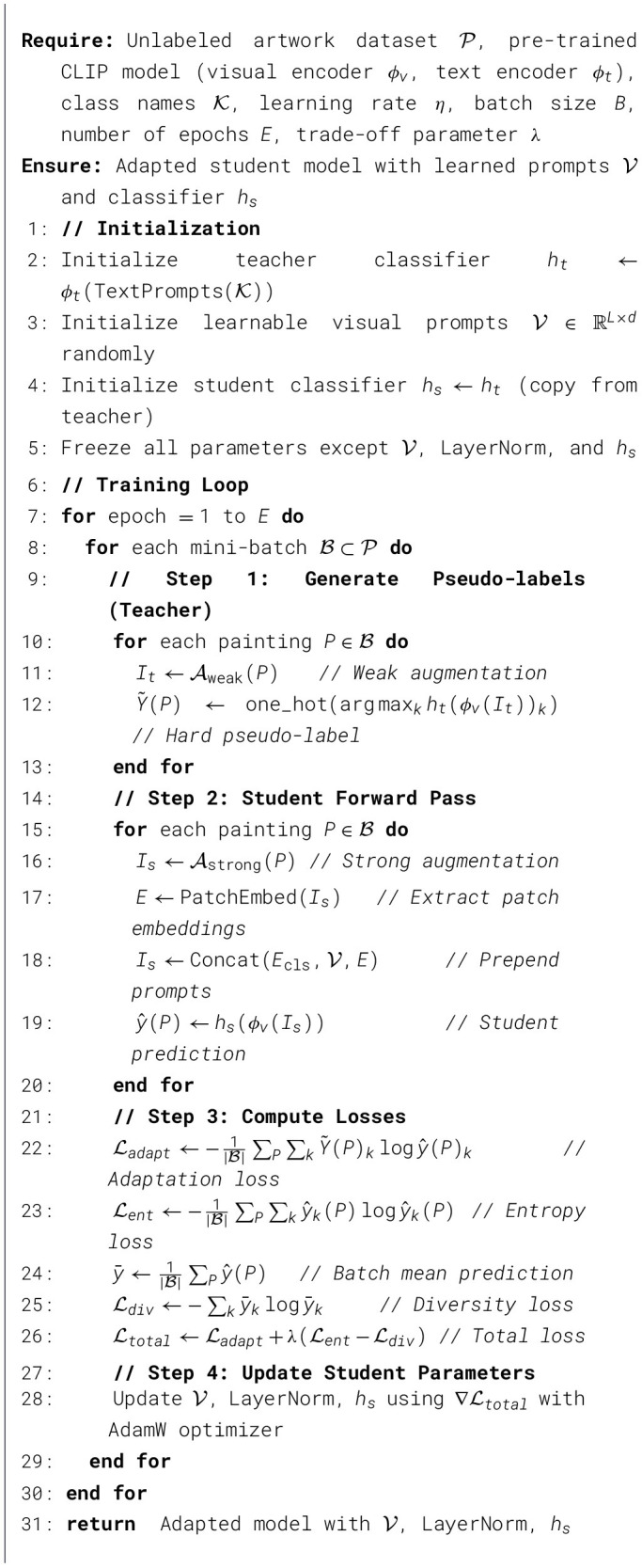

## 4 Results

### 4.1 Experiment setup

#### 4.1.1 Dataset

To evaluate the performance of models in artistic contexts, we utilize two stylized variants of the Imagenet dataset: Imagenet-Sketch and Imagenet-Rendition. These datasets are specifically designed to simulate artistic representations by introducing significant deviations in visual style compared to natural images.

Imagenet-Sketch: Imagenet-Sketch (Wang et al., 2019) is a benchmark dataset designed to evaluate the generalization ability of visual recognition models under significant domain shifts. It comprises 50,000 hand-drawn sketches that span 1,000 object categories aligned with those in the original Imagenet dataset. Unlike natural images, these sketches are grayscale and devoid of texture, emphasizing structural features such as contours and shapes rather than color or fine-grained details. This deliberate abstraction simulates the characteristics of sketch-based representations commonly encountered in human-created artworks. As such, Imagenet-S serves as a challenging testbed for assessing a model's robustness to texture bias and its capacity to rely on high-level semantic abstractions when recognizing objects in stylized or out-of-distribution scenarios.

Imagenet-Rendition: Imagenet-Rn (Hendrycks et al., 2021) is a domain-shifted benchmark dataset consisting of approximately 54,000 images that depict objects from the original Imagenet classes rendered in various non-photorealistic styles. These include a wide range of artistic and synthetic modalities such as watercolor paintings, oil paintings, 3D computer-generated renderings, and handcrafted paper sculptures. The images exhibit significant deviations from standard natural photographs, both in terms of texture and visual style, thereby introducing substantial distributional shifts. Owing to its rich diversity of artistic abstractions, Imagenet-Rendition serves as a rigorous testbed for evaluating the robustness and generalization ability of image classification models when faced with substantial changes in visual appearance and domain characteristics.

In this section, we first describe the baseline models used for evaluation. We then present the experimental setup, including implementation details and evaluation metrics. Finally, we report and analyze the results of our proposed method compared to existing approaches. In our experiments, we use the standard class labels provided with each dataset without relabeling.

It is important to note that both Imagenet-Sketch and Imagenet-Rendition significantly diverge from the canonical Imagenet validation set in terms of texture, color, and overall visual composition, thereby offering a rigorous testbed for object recognition under stylized and artistic transformations. The dataset was randomly divided into 80% for training and 20% for testing to ensure a balanced evaluation protocol.

#### 4.1.2 Evaluation results

Baseline: we compare our method against several recent approaches for zero-shot and pseudo-labeled image classification:

- CLIP (Radford et al., 2021) performs zero-shot classification by measuring the cosine similarity between image and text embeddings extracted from frozen vision and language encoders.
- UPL (Zhou et al., 2022b) introduces learnable text prompts for the CLIP text encoder and updates them using confidence based pseudo labels, without requiring labeled training data.
- CLIP-PR (Li et al., 2023) extends CLIP by training an adapter on top of the vision encoder, leveraging label distribution priors from downstream datasets to generate more accurate pseudo-labels.
- LaFTer (Mirza et al., 2023) refines zero-shot classifiers using a collection of unlabeled images and language supervision from pre-trained language models, enabling label-free tuning without manual annotations.

#### 4.1.3 Implementation details

All experiments are implemented using the Dassl.pytorch framework and conducted on a single NVIDIA RTX 4090 GPU with 24GB of memory. The vision backbone is ViT-B/32, initialized from a pre-trained CLIP model, with frozen encoders during zero-shot inference and fine-tuning applied to the parameter-efficient module only. We adopt a batch size of 50 and utilize four data loading workers. The initial learning rate is set to 0.0005, with a weight decay of 1 × 10^−4^, and training is conducted for 10 epochs, visual prompt num set to *N* = 50. Optimization is performed using the Adam W optimizer. Mixed-precision training (fp16) is employed throughout to accelerate computation and reduce memory consumption.

### 4.2 Main results

To validate the effectiveness of our proposed InfoMSD approach for artwork recognition, we conduct comprehensive experiments following the evaluation setup described above.

Our evaluation focuses on two key aspects: (1) quantitative performance comparison with state-of-the-art zero-shot and domain adaptation methods on standard benchmarks, and (2) qualitative analysis of the learned representations and their transferability to artistic domains. The experiments are designed to demonstrate how information maximization principles, enable effective knowledge transfer from natural image domains to artwork without requiring labeled artistic data.

The main quantitative results are presented in Tables 1, 2. As shown in Table 1, our InfoMSD method achieves state-of-the-art performance on both benchmarks, reaching 73.24% accuracy on Imagenet-Rendition and 44.14% on Imagenet-Sketch. This represents a significant improvement of 1.35 and 0.96% over the strong LaFTer baseline, respectively. The substantial gains over all competing methods validate the effectiveness of our proposed self-distillation framework augmented with information-theoretic regularization.

**Table 1:** Performance comparison on Imagenet-Rendition and Imagenet-Sketch (%).

**Method**	**Imagenet-Rendition**	**Imagenet-Sketch**
CLIP	66.81	41.12
CLIP-PR	54.27	38.95
UPL	65.66	42.40
LaFTer	71.89	43.18
**InfoMSD (Ours)**	**73.24**	**44.14**

Bold values indicate the best performance.

**Table 2:** Imagenet-Rendition and Imagenet-Sketch Top-k ACC comparison.

**Method**	**TOP1**	**TOP5**	**TOP10**	**TOP50**	**TOP100**
**Imagenet-Rendition**					
CLIP	66.81	86.83	91.42	98.01	99.37
LaFTer	71.89	90.61	94.20	98.57	99.55
**InfoMSD**	**73.24**	**91.31**	**94.67**	**98.71**	**99.61**
**Imagenet-Sketch**					
CLIP	41.12	69.21	78.38	91.13	94.27
LaFTer	43.18	69.60	78.14	91.06	94.32
**InfoMSD**	**44.14**	**71.19**	**79.70**	**92.16**	**95.09**

Bold values indicate the best performance.

Furthermore, the Top-k accuracy results in Table 2 offer a more holistic view of our model's performance. Beyond the established Top-1 superiority, InfoMSD consistently maintains or extends its lead over LaFTer across the Top-5 and Top-10 metrics on both datasets. This consistent out-performance across the entire prediction spectrum strongly suggests that our information maximization objective does more than just boost the top prediction; it enhances the overall quality of the feature representations, leading to a more semantically coherent ranking of all potential classes.

In summary, the consistent and significant improvements across these challenging artistic-style benchmarks strongly support our hypothesis that integrating self-distillation with information maximization is a highly effective strategy for adapting vision-language models to non-photographic domains like artwork, all without requiring any labeled artistic data.

### 4.3 Comparison with few-shot learning methods

**Figure 3:**
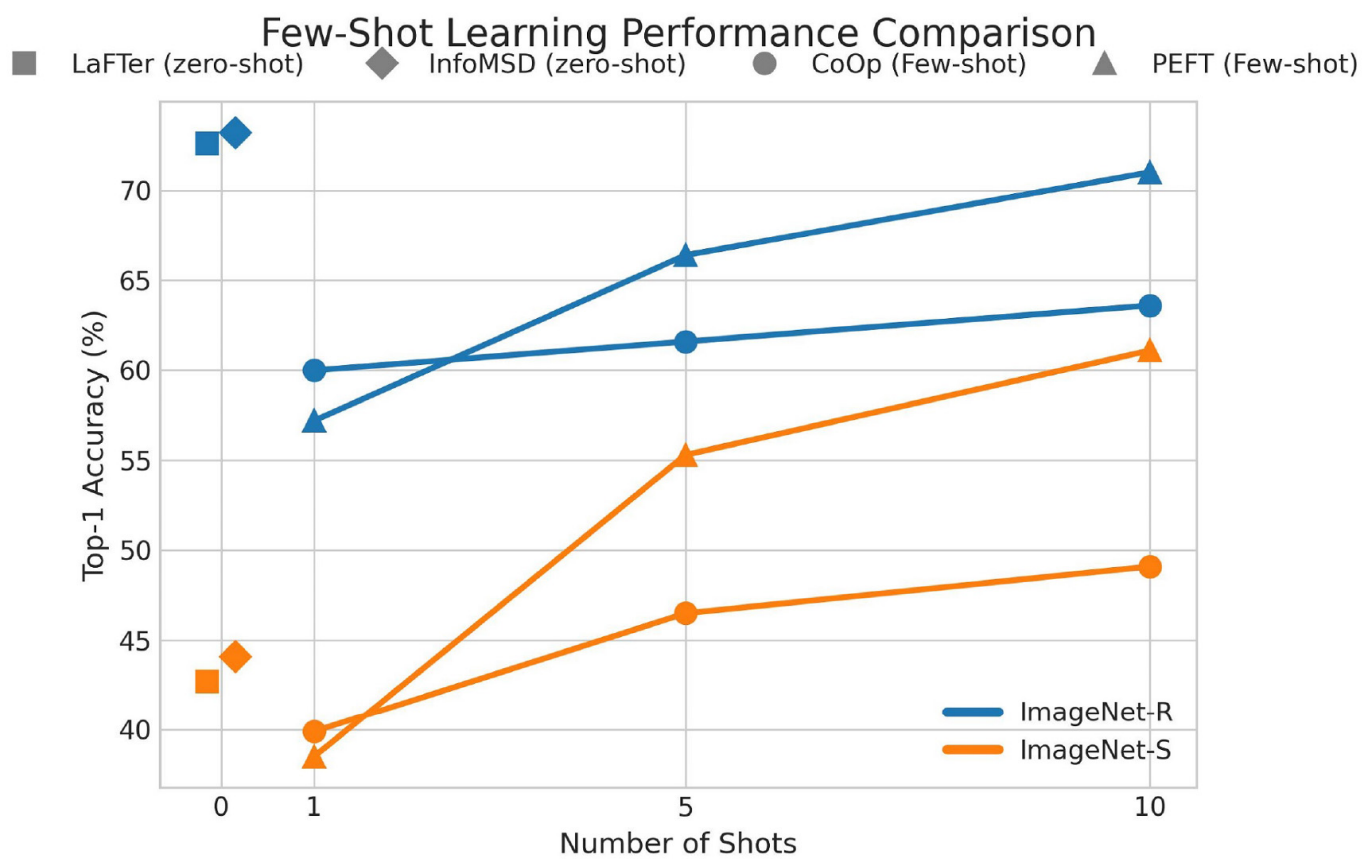
Comparison of zero-shot (InfoMSD, LaFTer) and few-shot (CoOp, PEFT) methods on Imagenet-Rendition and Imagenet-Sketch. We plot Top-1 accuracy against the number of training shots, with our method serving as a *k* = 0 baseline.

To further contextualize the performance of our zero-shot method, we benchmark InfoMSD against prominent few-shot learning paradigms, namely CoOp and Parameter-Efficient Fine-Tuning (PEFT). Figure 3 illustrates this comparison on the Imagenet-R and Imagenet-S datasets. The plot shows the Top-1 accuracy as a function of the number of training shots (*k*), where our method and LaFTer serve as zero-shot baselines (*k = 0*) against CoOp and PEFT, which are evaluated at *k* = {1, 5, 10}.

The results yield several key insights. At the zero-shot level, InfoMSD consistently outperforms the strong baseline LaFTer on both datasets, achieving 73.2% on Imagenet-R and 44.1% on Imagenet-S. More notably, on Imagenet-R, our zero-shot model's performance surpasses that of both CoOp and PEFT even at 10 shots, demonstrating its exceptional generalization capability and efficiency. On the more challenging Imagenet-S, while PEFT's performance scales with more data and eventually exceeds the zero-shot baseline after five shots, InfoMSD still provides a stronger starting point than 1-shot learners. This analysis underscores that InfoMSD serves as a highly competitive baseline, challenging the necessity of few-shot training in certain low-data scenarios.

### 4.4 Feature and output analysis

On the Imagenet-Rendition dataset, the CLIP baseline yields feature clusters that are largely entangled, with considerable overlap and poorly defined inter-class boundaries. This suggests a limited ability to separate object categories when exposed to non-photorealistic, stylistically diverse inputs. In contrast, InfoMSD produces feature embeddings that are more clearly partitioned across categories, exhibiting enhanced inter-class separability and more compact intra-class cohesion. These properties reflect a stronger alignment between semantic similarity and geometric proximity in the learned feature space.

A similar pattern is observed on the Imagenet-Sketch dataset. While CLIP features tend to form diffuse groupings with fuzzy class boundaries, InfoMSD representations demonstrate tight, well-defined clusters corresponding to individual categories. The categorical structure is more pronounced and robust to the lack of texture and color, which are characteristic challenges of sketch-based imagery.

**Figure 4:**
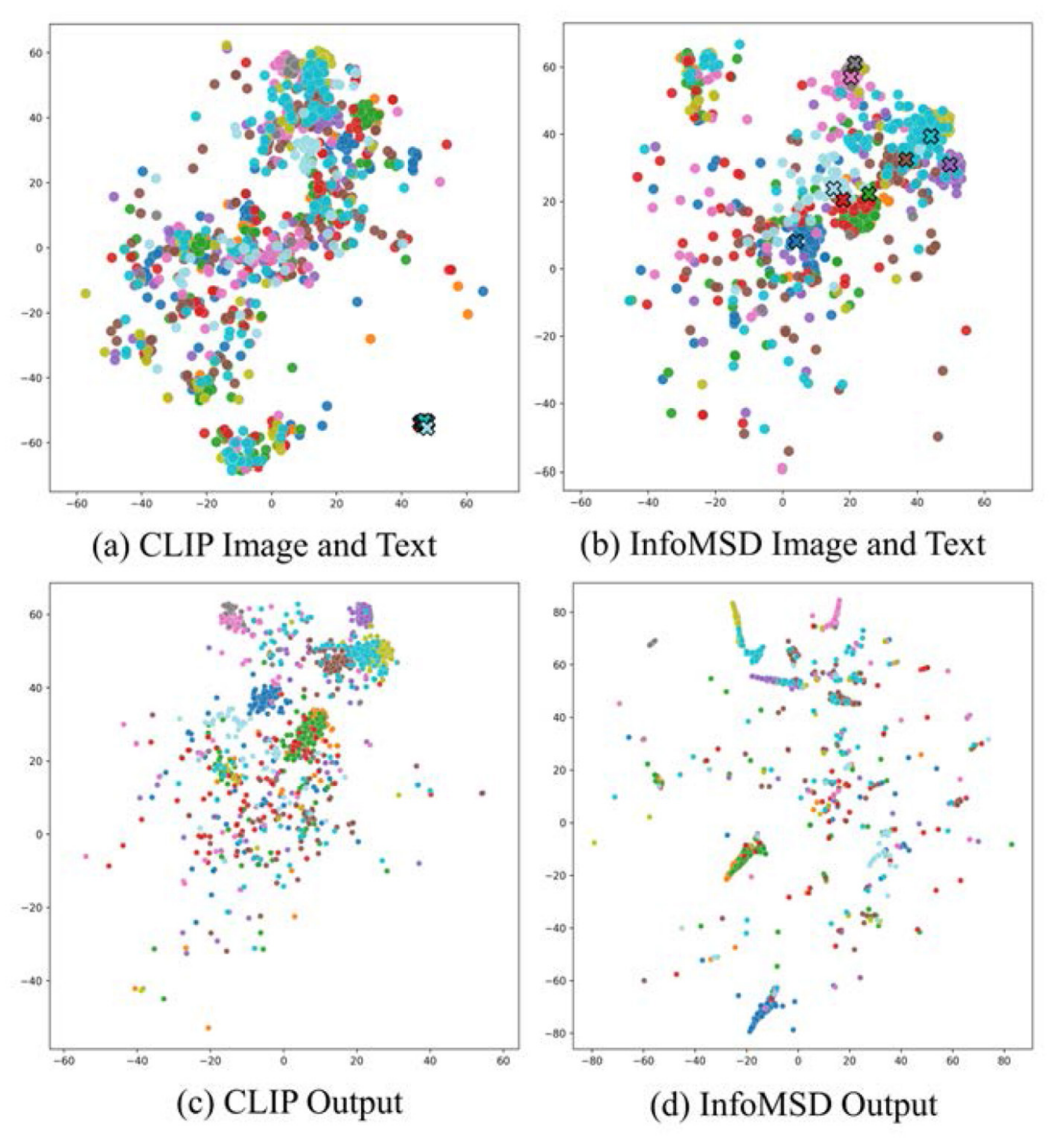
t-SNE visualization on Imagenet Sketch: **(a, b)** feature representations; **(c, d)** output distributions. InfoMSD shows better inter-class separation than CLIP. Circles (°): images; crosses (×): text.

Overall, the superior clustering quality of InfoMSD in both scenarios highlights the efficacy of our information maximization strategy. It enables the model to extract semantically meaningful and visually discriminative representations under large domain shifts, thereby supporting improved generalization to artistic and abstract visual domains. Figure 4 presents the t-SNE visualizations of the learned feature representations obtained from the CLIP baseline and our proposed *InfoMSD* method, evaluated on two domain-shifted datasets: Imagenet-Rendition and Imagenet-Sketch. These visualizations provide intuitive insights into the discriminative capacity and structural organization of the feature spaces induced by the two approaches.

### 4.5 Ablation study

Having demonstrated the superior performance of our InfoMSD method against existing approaches, we now turn to a detailed analysis of the individual components that contribute to its effectiveness. The strong performance gains observed in Table 1 raise important questions about which specific design choices and theoretical principles drive these improvements. To provide deeper insights into our method's success and validate the necessity of each component, we conduct a comprehensive ablation study. The ablation analysis is particularly crucial for understanding how information maximization principles translate into practical performance gains in the artwork domain.

By systematically removing key components—including the adapter modules, layer normalization, visual prompt tuning, and entropy maximization can isolate the contribution of each element and justify our architectural decisions. This analysis not only validates our design choices but also provides guidance for future work in cross-domain object recognition. Table 3 presents the ablation study analyzing individual components of our InfoMSD framework. We systematically remove key components to validate their contributions.

**Table 3:** Component ablation study on Imagenet-Rendition and Imagenet-Sketch (%).

**Method**	**Imagenet-Renditio**	**Imagenet-Sketchs**
w/o adapter	72.01	40.77
w/o LayerNorm	69.68	41.85
w/o VPT	53.09	14.76
w/o Entropy	70.34	42.24
w/o Divergence	72.26	42.46
InfoMSD	**73.24**	**44.14**

Bold values indicate the best performance.

The results demonstrate that each component contributes meaningfully to the final performance.

Parameter-efficient fine-tuning: disabling Visual Prompt Tuning causes the most significant performance degradation (20.15% on Imagenet-R and 29.38% on Imagenet-S), underscoring its indispensable role in adapting the model's feature space to artistic domains. Fine-tuning LayerNorm also proves crucial, as its removal leads to a notable drop (e.g., 3.56% on Imagenet-R), highlighting the importance of stabilizing feature statistics during domain shifts.

Information-theoretic regularization: both components of our information maximization loss are vital for refining performance. Removing the conditional entropy minimization (“w/o Entropy”) term, which encourages prediction confidence, results in a 2.90% drop on Imagenet-R. Similarly, removing the diversity maximization (“w/o Divergence”) term, which prevents mode collapse, leads to a 1.00% drop.

To further validate the generalization of our InfoMSD approach, we evaluate its performance across different Vision Transformer backbones of varying scales. This analysis demonstrates the architectural flexibility of our information maximization framework and reveals how the benefits scale with model capacity. Table 4 compares different Vision Transformer backbones to evaluate the scalability of our approach.

**Table 4:** Performance comparison of different backbone architectures on ImageNet-R and ImageNet-S.

**Backbone**	**Method**	**Imagenet-R**	**Imagenet-S**
ViT-B32	CLIP	66.81	41.12
	LaFTer	71.89	43.18
	InfoMSD	**73.24**	**44.14**
ViT-B16	CLIP	73.78	46.61
	LaFTer	79.99	49.48
	InfoMSD	**81.08**	**50.21**
ViT-L14	CLIP	85.27	58.04
	LaFTer	86.54	60.72
	InfoMSD	**88.89**	**61.17**

Bold values indicate the best performance.

Our method consistently outperforms baselines across all backbone architectures. The performance gains are maintained as model capacity increases, with ViT-L14 achieving 88.89% on Imagenet-R and 61.17% on Imagenet-S. This demonstrates the scalability and robustness of our information maximization approach.

### 4.6 Training efficiency analysis

While performance improvements are crucial, practical deployment of domain adaptation methods also depends heavily on training efficiency and computational feasibility. Having established the effectiveness and generalization of our InfoMSD approach, we now examine its practical advantages in terms of parameter efficiency and computational requirements. Table 5 demonstrates the training efficiency of our InfoMSD method, highlighting its parameter efficiency and computational advantages.

**Table 5:** Training efficiency comparison showing model parameters, update requirements, and computational costs.

**Dataset**	Model parameters			Model update			Training efficiency		
	**Visual (VPT, LN)**	**Textual (Adapter)**	**Trainable params**	**Total params**	**Update ratio**	**Time/ epoch**	**Total time**	**GPU mem**	**CPU mem**
Imagenet-R	78.44K	102K	180.44K	~150M	0.12%	2 m 03 s	21 m 37 s	5.75GB	6.14GB
Imagenet-S	78.44K	512K	590.44K	~150M	0.39%	3 m 42 s	38 m 46 s	5.73GB	6.26GB

Our method achieves remarkable parameter efficiency by updating only 0.12 and 0.39% of total model parameters on Imagenet-R and Imagenet-S respectively. The trainable parameters consist primarily of the VPT tokens, layer normalization modules, and lightweight text adapters, totaling less than 600K parameters across both datasets. The training efficiency is further demonstrated by the modest computational requirements. Training completes within 22–39 min with GPU memory usage under 6GB, making our approach highly practical for deployment scenarios with limited computational resources. Building upon LaFTer's label-free foundation, our InfoMSD method not only eliminates the need for annotated data but also significantly reduces parameter overhead and training time, establishing a highly efficient framework for artwork recognition.

### 4.7 Verification on natural datasets

To rigorously evaluate the generalization capabilities and robustness of our proposed method, InfoMSD, we conducted extensive experiments on a diverse benchmark of 11 publicly available datasets. This benchmark suite was curated to span a wide spectrum of visual recognition challenges, moving beyond the initial validation on Imagenet-R and Imagenet-S. The datasets encompass tasks in general object recognition [e.g., CIFAR-10/100 (Krizhevsky, 2009), Caltech-101 (Fei-Fei et al., 2004)], fine-grained visual classification [e.g., Oxford-Pets (Parkhi et al., 2012), Aircraft (Maji et al., 2013), Food-101 (Bossard et al., 2014), Flower (Nilsback and Zisserman, 2008)], and specialized domains such as satellite imagery (Eurosat) (Helber et al., 2019), texture analysis (DTD) (Cimpoi et al., 2014), scene recognition (SUN397) (Xiao et al., 2010), and action recognition (UCF101) (Soomro et al., 2012). A detailed description of each dataset is provided below:

General object recognition
  - CIFAR-10: comprises 60,000 color images (32 × 32 pixels) across 10 common object classes, with 6,000 images per class.
  - CIFAR-100: a more challenging version of CIFAR-10, featuring 60,000 images distributed over 100 fine-grained classes (600 images per class).
  - Caltech-101: a collection of 9,144 images belonging to 101 object categories, with a variable number of images per category.
Fine-grained visual classification
  - Oxford-Pet (Pets): features 7,349 images of 37 cat and dog breeds, with approximately 200 images per breed.
  - FGVC-Aircraft (Aircraft): contains 10,200 images of 100 different aircraft model variants (100 images each).
  - Food-101: a large-scale dataset with 101,000 images of 101 food categories, containing 1,000 images per category.
  - Oxford-Flower (Flower): consists of 8,189 flower images from 102 different species, with image counts per species ranging from 40 to 258.
Specialized domain classification
  - Eurosat: a dataset of 27,000 satellite images for land use and land cover classification, divided into 10 categories.
  - Describable textures dataset (DTD): includes 5,640 texture images across 47 categories, with 120 images for each.
  - SUN397: a comprehensive scene recognition dataset with over 100,000 images from 397 distinct scene types.
  - UCF101: an action recognition dataset containing 13,320 video clips from 101 action categories. For our experiments, representative frames are extracted for static image classification.

We benchmark InfoMSD against the foundational zero-shot CLIP model. The detailed performance metrics, including Top-k accuracy, are presented in Tables 6, 7. The results unequivocally demonstrate the superiority of our method. As shown in the tables, InfoMSD consistently outperforms the CLIP baseline across nearly all datasets and metrics.

**Table 6:** Performance comparison on smaller-scale datasets (Part 1).

**Dataset (Classes)**	**TOP1**	**TOP5**	**TOP10**	**TOP25**
CIFAR-10 (10)	88.76 / 95.87	99.38 / 99.96	—	—
	(**+7.11**)	(**+0.58**)	—	—
Eurosat (10)	35.57 / 79.70	84.62 / 97.59	—	—
	(**+44.13**)	(**+12.97**)	—	—
Pets (37)	79.97 / 89.43	95.64 / 99.76	96.92 / 99.95	99.81 / 100.00
	(**+9.46**)	(**+4.12**)	(**+3.03**)	(**+0.19**)
DTD (47)	43.62 / 53.01	72.46 / 84.63	84.57 / 93.97	96.39 / 99.47
	(**+9.39**)	(**+12.17**)	(**+9.40**)	(**+3.08**)

Results are presented in the format: CLIP / InfoMSD. The second row for each dataset shows the performance change. The color values indicate performance changes (Our method vs. CLIP). Green represents an increase in value, while red represents a decrease in value.

**Table 7:** Performance comparison on larger-scale datasets (Part 2).

**Dataset (Classes)**	**TOP1**	**TOP5**	**TOP10**	**TOP50**	**TOP75 / 100**
CIFAR-100 (100)	64.23 / 74.95	88.14 / 94.94	93.35 / 97.58	99.17 / 99.84	99.76 / 99.96
	(**+10.72**)	(**+6.80**)	(**+4.23**)	(**+0.67**)	(**+0.20**)
Aircraft (101)	17.67 / 21.33	48.90 / 46.64	66.79 / 63.07	97.27 / 97.93	99.01 / 99.52
	(**+3.66**)	(−2.26)	(−3.72)	(**+0.66**)	(**+0.51**)
Food101 (102)	76.97 / 80.63	94.99 / 95.97	97.60 / 98.06	99.75 / 99.78	99.92 / 99.93
	(**+3.66**)	(**+0.98**)	(**+0.46**)	(**+0.03**)	(**+0.01**)
Flower (102)	63.74 / 73.12	84.53 / 88.75	87.98 / 91.27	96.10 / 98.66	98.74 / 99.96
	(**+9.38**)	(**+4.22**)	(**+3.29**)	(**+2.56**)	(**+1.22**)
UCF101 (101)	60.16 / 68.91	84.46 / 92.04	91.17 / 96.88	99.31 / 99.97	100.00 / 100.00
	(**+8.75**)	(**+7.58**)	(**+5.71**)	(**+0.66**)	(0.00)
Caltech101 (100)	90.59 / 93.71	99.39 / 99.96	99.80 / 100.00	100.00 / 100.00	100.00 / 100.00
	(**+3.12**)	(**+0.57**)	(**+0.20**)	(0.00)	(0.00)
SUN397 (397)	60.26 / 66.50	89.47 / 92.27	94.61 / 96.26	99.11 / 99.42	99.66 / 99.81
	(**+6.24**)	(**+2.80**)	(**+1.65**)	(**+0.31**)	(**+0.15**)

Results are presented in the format: CLIP / InfoMSD. The second row shows the performance change. The final column reports Top-75 accuracy, except for SUN397 which reports Top-100. The color values indicate performance changes (Our method vs. CLIP). Green represents an increase in value, while red represents a decrease in value.

On the smaller-scale datasets (Table 6), the performance gains are particularly striking. For instance, InfoMSD achieves a remarkable +44.13% increase in Top-1 accuracy on Eurosat, boosting performance from 35.57 to 79.70%. Substantial improvements are also observed on CIFAR-10 (+7.11%), Oxford-Pets (+9.46%), and DTD (+9.39%), highlighting our method's efficacy on tasks with both coarse and fine-grained distinctions in a limited class setting.

**Figure 5:**
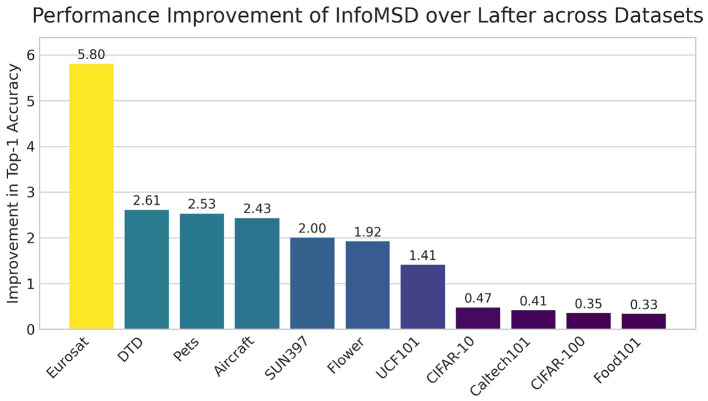
Top-1 accuracy improvement of InfoMSD over the LaFTer baseline across 11 datasets. The bar chart displays the absolute performance gain (%), with datasets sorted by the magnitude of the improvement.

A direct comparison against the competitive LaFTer baseline was conducted to quantify the performance advantage of InfoMSD. Figure 5 visualizes the absolute improvement in Top-1 accuracy on all 11 datasets, which are sorted in descending order of the performance gain for clarity. The results demonstrate a consistent and often substantial advantage for our method. The most significant gain is observed on Eurosat, with a remarkable +5.80% improvement, underscoring InfoMSD's efficacy in specialized domains. Substantial improvements exceeding 2.0% are also registered on other challenging datasets, including fine-grained classification tasks such as DTD (+2.61%), Pets (+2.53%), and Aircraft (+2.43%), as well as the scene recognition benchmark SUN397 (+2.00%). While the gains are more modest on general object recognition datasets like CIFAR-10 (+0.47%) and Food101 (+0.33%)—where baseline performance is already high—InfoMSD still maintains a clear and positive margin. Crucially, the universal outperformance across every dataset, irrespective of its domain or complexity, provides compelling evidence of InfoMSD's superior robustness and generalization capabilities compared to LaFTer.

In summary, the comprehensive evaluation across these 11 diverse datasets provides compelling evidence of InfoMSD's effectiveness. The consistent and often substantial improvements over both the foundational CLIP model and a competitive method like LaFTer underscore its superior generalization ability for zero-shot classification tasks.

## 5 Discussion

The InfoMSD method exhibits several inherent limitations that warrant discussion. First, the approach requires simultaneous optimization of information maximization objectives and cross-modal alignment, resulting in higher computational overhead compared to traditional uni-modal approaches. This increased complexity may present efficiency bottlenecks when processing large-scale artwork datasets. Additionally, due to the adoption of unsupervised domain adaptation with information maximization strategies, the model performance exhibits sensitivity to initial parameter settings, where different initializations may lead to convergence to distinct local optima, affecting stability and reproducibility. Furthermore, while the method demonstrates superior performance in artwork recognition, its generalization capability to other artistic styles such as contemporary abstract art or digital art requires further empirical validation.

### 5.1 Visual interpretability methods for vision-language models

Interpreting deep neural networks is essential for building trust, ensuring accountability, and diagnosing model behavior, especially in safety-critical or domain-shifted scenarios. In this section, we review four widely-used *post-hoc* visual explanation techniques—Grad-CAM, LIME, SHAP, and MM-SHAP—that aim to attribute model predictions to input features or modalities. These methods differ in their theoretical assumptions, applicability to model architectures, and granularity of interpretation. We present their underlying mathematical formulations and highlight how each captures distinct perspectives on model behavior, ranging from spatial saliency (Grad-CAM) to feature and modality contributions (SHAP, MM-SHAP).

Grad-CAM: Gradient-weighted Class Activation Mapping (Selvaraju et al., 2017) highlights salient regions by weighting feature maps with class-specific gradients. Given a target class score *y*^*c*^ and activation maps *A*^*k*^∈ℝ^*H*×*W*^, the importance of channel *k* is:


$$\begin{array}{l} \alpha_{k}^{c}=\frac{1}{Z}\sum_{i,j}\frac{\partial y^{c}}{\partial A_{ij}^{k}} \end{array}$$ (10)

where *Z* = *H*×*W*. These weights modulate the activation maps to produce the Grad-CAM heatmap:


$$\begin{array}{l} L^{c}=\text{ReLU}\left(\sum_{k}\alpha_{k}^{c}A^{k}\right) \end{array}$$ (11)

This results in a class-discriminative saliency map localized in the input space.

LIME: Local Interpretable Model-agnostic Explanations (Ribeiro et al., 2016) approximates the decision boundary of a black-box model *f* around an input *x* using an interpretable model *g*. It perturbs *x* to form samples $\left\{x_{i}^{\prime }\right\}$, then solves:


$$\begin{array}{l} \xi \left(x\right)=\arg\underset{g\in G}{\min}\sum_{i}\pi_{x}\left(x_{i}^{\prime }\right){\left(f\left(x_{i}^{\prime }\right)-g\left(x_{i}^{\prime }\right)\right)}^{2}+\Omega \left(g\right) \end{array}$$ (12)

Here, $\pi_{x}\left(x^{\prime }\right)$ defines locality via a kernel (e.g., cosine or exponential distance), and Ω(*g*) encourages sparsity for interpretability. The learned *g* is then interpreted to explain *f*(*x*).

SHAP: Shapley Additive Explanations (Mosca et al., 2022) assigns each feature *i* a value ϕ_*i*_ representing its average marginal contribution across all feature coalitions:


$$\begin{array}{l} \phi_{i}=\sum_{S\subseteq F\backslash \left\{i\right\}}w\left(S\right)\left[f\left(S\cup \left\{i\right\}\right)-f\left(S\right)\right] \end{array}$$ (13)

where $w\left(S\right)=\frac{|S|!\left(|F|-|S|-1\right)!}{|F|!}$. In practice, SHAP approximates this via sampling:


$$\begin{array}{l} \phi_{i}\approx E_{S}\left[f\left(S\cup \left\{i\right\}\right)-f\left(S\right)\right] \end{array}$$ (14)

This guarantees consistency and local accuracy under mild conditions.

MM-SHAP: performance-agnostic Metric for Measuring Multimodal Contributions (Parcalabescu and Frank, 2023) generalizes SHAP to multimodal inputs. Let *M* = {*m*_1_, …, *m*_*K*_} be the modalities. The Shapley value of modality *m*_*i*_ is:


$$\begin{array}{l} \phi_{m_{i}}=\sum_{S\subseteq M\backslash \left\{m_{i}\right\}}w\left(S\right)\left[f\left(S\cup \left\{m_{i}\right\}\right)-f\left(S\right)\right] \end{array}$$ (15)

with $w\left(S\right)=\frac{|S|!\left(K-|S|-1\right)!}{K!}$. A Monte Carlo estimate is:


$$\begin{array}{l} \phi_{m_{i}}\approx E_{S}\left[f\left(S\cup \left\{m_{i}\right\}\right)-f\left(S\right)\right] \end{array}$$ (16)

This quantifies each modality's contribution, independent of accuracy, enabling fine-grained attribution in vision-language systems.

### 5.2 Why are multimodal methods better than single-modality in artwork recognition?

We employ *open-source* explanation tools—Grad-CAM, LIME, SHAP, and MM-SHAP—*solely* to analyze and illustrate the behavior of a pretrained multimodal model (CLIP). These tools are not proposed by this work; they are used to expose how multimodal alignment manifests in practice.

#### 5.2.1 From visuals to evidence

**Figure 6:**
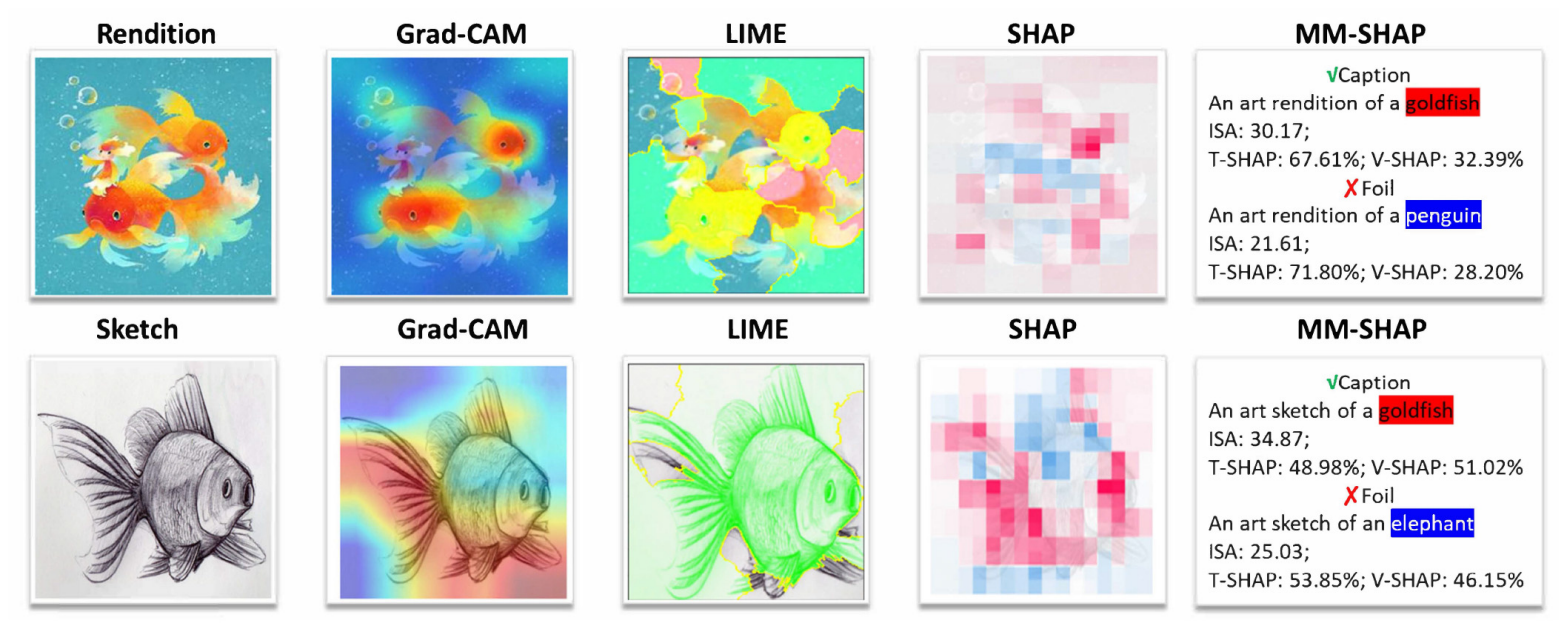
Open-source explanations evidencing multimodal benefits (methods not proposed here). For both rendition and sketch styles, Grad-CAM localizes the goldfish body; LIME selects sparse, object-relevant superpixels; SHAP yields consistent positive attributions along object contours. MM-SHAP further quantifies cross-modal consistency: the true caption “goldfish” attains higher image-sentence alignment (ISA; e.g., 30.17 for rendition and 34.87 for sketch) than foils (“penguin”/“elephant”), revealing textual counterfactual sensitivity. The modality contributions adapt with style: higher T-SHAP in rendition (e.g., 67.61%) and more balanced T/V-SHAP in sketch (e.g., 48.98%), indicating stronger reliance on visual evidence under abstraction. Together, these visuals provide convergent support from location, feature importance, semantic alignment, and modality allocation.

Our claim that multimodal models surpass unimodal ones is supported—without introducing new experiments—by four complementary lines of evidence observed in Figure 6.

(E1) Textual counterfactual sensitivity (real use of the second modality). Holding the image fixed while replacing the true caption *goldfish* with foils (*penguin*/*elephant*) consistently lowers the Image-Sentence-Alignment (ISA), with the true caption remaining higher (e.g., 30.17/34.87 vs. markedly smaller for foils). If the model did not exploit text, such systematic ISA separation would not occur; hence the text modality is *functionally* engaged.

(E2) Cross-method consistency (non-accidental evidence). Grad-CAM highlights the goldfish body, LIME selects object-coherent superpixels, and SHAP concentrates positive contributions along semantic contours. MM-SHAP integrates these visual cues with text by summarizing alignment (ISA) and per-modality contributions (T-/V-SHAP). Agreement across heterogeneous tools—spatial saliency, feature-level attribution, and multimodal attribution—reduces the risk of explanation idiosyncrasies and strengthens interpretive confidence.

(E3) Modality adaptation (a robustness mechanism). In rendition images, T-SHAP is higher, indicating stronger use of textual priors; in sketch images, V-SHAP rises and the contributions become more balanced. This *adaptive reallocation* of evidence between text and vision is a capability unimodal models lack, and it explains why semantic alignment remains stable when visual detail is abstracted.

(E4) Semantic alignment rather than mere saliency. Unimodal heatmaps primarily reflect where the image is visually striking, not *why* a specific concept is predicted. By directly relating image regions to caption semantics, MM-SHAP and ISA target the task objective: the true caption aligns with the object while foils weaken alignment. Thus, multimodal explanations answer the semantic “why” beyond highlighting the visually conspicuous.

The explainability analysis in Section 5.2 directly validates InfoMSD's core mechanisms. First, the entropy minimization term ($L_{\text{ent}}$) encourages confident predictions, which manifests as focused Grad-CAM heatmaps concentrated on semantically relevant regions (e.g., the goldfish body) rather than scattered attention. Second, the diversity loss ($L_{\text{div}}$) maintains discriminative class boundaries, enabling meaningful MM-SHAP counterfactual analysis—swapping “goldfish” with “penguin” produces substantial ISA drops because the model preserves distinct class representations. Third, the adaptive T-SHAP/V-SHAP modality weighting (higher text reliance for renditions, more balanced for sketches) reflects how our visual prompts learn domain-specific compensations. Together, these observations confirm that InfoMSD's information-theoretic objectives translate into interpretable and trustworthy decision-making patterns for artwork recognition.

#### 5.2.2 Robustness under style shift

Sketch inputs dilute texture and edges, making pure vision cues less separable (LIME regions become less distinct; SHAP exhibits higher noise). Yet the multimodal readout in Figure 6 maintains a higher ISA for the true caption and rebalances T/V contributions. When visual evidence weakens, textual semantics and cross-modal alignment compensate, yielding more stable decisions than unimodal alternatives.

Without adding experiments, the existing visuals already constitute a coherent argument: (i) textual counterfactuals modulate alignment, evidencing genuine use of text; (ii) diverse explanation tools converge on the same object and concept; (iii) modality contributions adapt to input style, providing a plausible robustness mechanism; and (iv) alignment measures capture *semantic* consistency rather than saliency alone. Collectively, these observations explain *why* multimodal modeling offers superior interpretability and stability over single-modality recognition in our setting.

### 5.3 Limitations on benchmark datasets

A further limitation concerns the scope of our experimental benchmarks. One reviewer suggested evaluating on additional domain generalization datasets such as PACS. In our study, we chose LaFTer as the primary baseline due to its significant influence as a state-of-the-art label-free adaptation method for vision-language models in recent years. However, LaFTer's framework requires dataset-specific text corpus generation through large language models (LLMs) to construct domain-relevant textual knowledge for each benchmark. Unfortunately, LaFTer does not provide pre-generated text corpora for datasets like PACS or WikiArt. Furthermore, publicly available museum-curated datasets with object-level annotations remain scarce, as most existing art datasets (e.g., WikiArt, Pandora-18K) focus on style or artist classification rather than semantic object recognition. If we were to independently generate text corpora for these datasets, it could introduce inconsistencies and potentially unfair comparisons, as the quality and coverage of LLM-generated descriptions may vary depending on prompt engineering and generation strategies.

We acknowledge this as a limitation of the current work. In future studies, we plan to (1) develop a standardized protocol for generating text corpora across diverse artistic datasets to ensure fair comparisons, (2) extend our evaluation to include style-oriented benchmarks such as WikiArt and Pandora-18K, and (3) explore hybrid recognition systems that jointly model both semantic content and artistic style attributes. We sincerely appreciate the reviewer's constructive suggestion, which will guide the direction of our follow-up research.

## 6 Conclusions

This study presents InfoMSD, a novel framework for object recognition in artwork that integrates information maximization with self-distillation under a zero-shot vision-language paradigm. By addressing the domain gap between natural and artistic images, InfoMSD enables more accurate representation learning in the absence of labeled training data.

The proposed method leverages vision-language priors and enhances them through an unsupervised optimization strategy that emphasizes discriminative feature extraction tailored to artistic imagery. Extensive experiments across multiple painting datasets demonstrate that InfoMSD consistently outperforms baseline methods, including CLIP, CLIP-PR, UPL, and LaFTer, particularly in cases involving stylistic abstraction and underrepresented object categories.

Beyond empirical performance, this work contributes a domain-adaptive approach that bridges computer vision and art analysis. It establishes a foundation for further research into explainable and culturally informed recognition systems, advancing the broader field of computational art understanding.

Furthermore, future work will explore scene-level understanding by modeling spatial and compositional relationships among multiple objects within a painting. This includes parsing visual hierarchies, spatial attention, and narrative structures embedded in complex scenes. Finally, we aim to develop interpretable models that facilitate collaboration with art historians. This includes employing explainable AI techniques such as attention-based saliency maps, concept attribution, and counterfactual generation to enhance transparency and domain trust in art-specific recognition systems.

## Data Availability

The datasets presented in this study can be found in online repositories. The names of the repository/repositories and accession number(s) can be found in the article/supplementary material.
